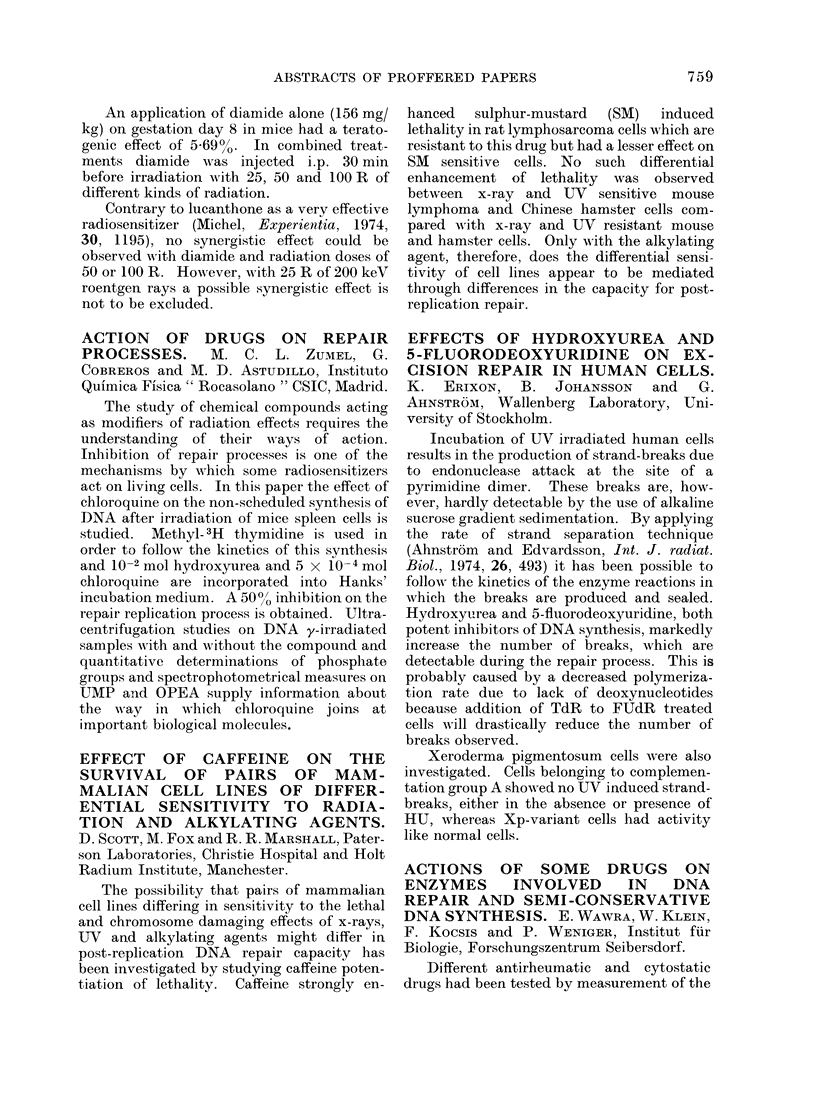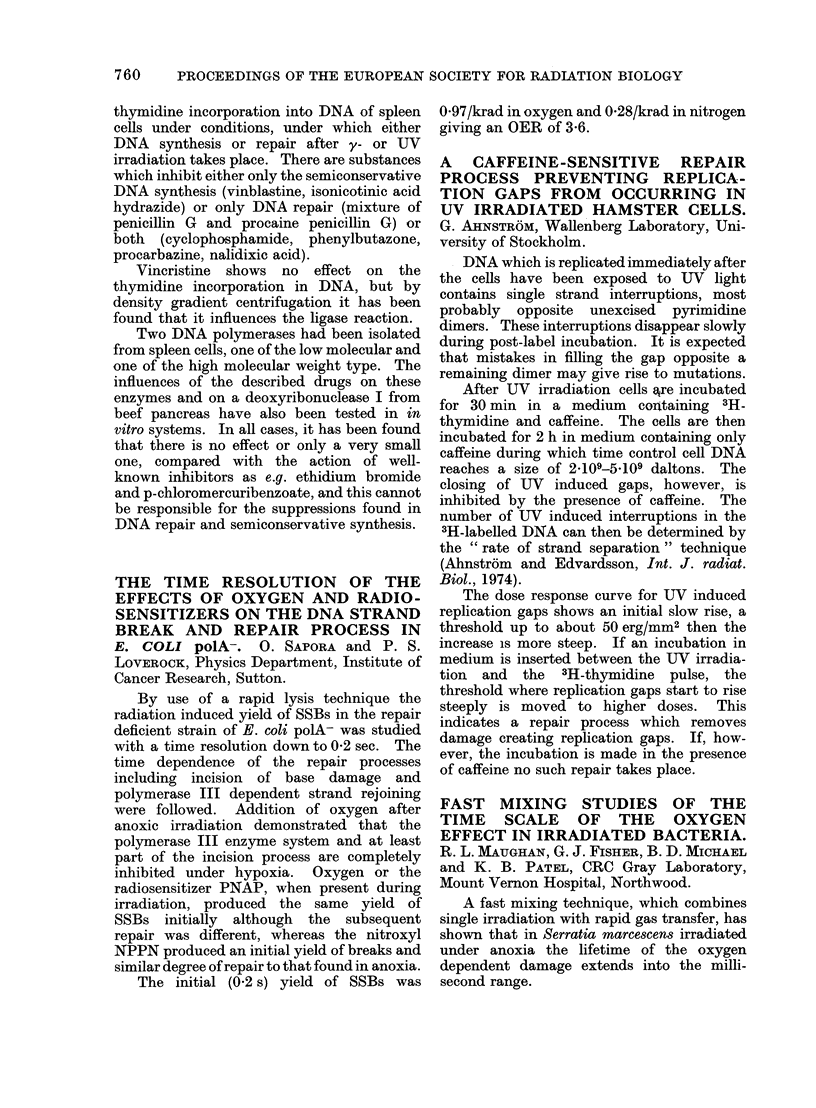# Proceedings: Actions of some drugs on enzymes involved in DNA repair and semi-conservative DNA synthesis.

**DOI:** 10.1038/bjc.1975.318

**Published:** 1975-12

**Authors:** E. Wawra, W. Klein, F. Kocsis, P. Weniger


**ACTIONS OF SOME DRUGS ON ENZYMES INVOLVED IN DNA REPAIR AND SEMI-CONSERVATIVE DNA SYNTHESIS.** E. Wawra, W. Klein, F. Kocsis and P. Weniger, Institut für Biologie, Forschungszentrum Seibersdorf.

Different antirheumatic and cytostatic drugs had been tested by measurement of the thymidine incorporation into DNA of spleen cells under conditions, under which either DNA synthesis or repair after $\gamma$- or UV irradiation takes place. There are substances which inhibit either only the semiconservative DNA synthesis (vinblastine, isonicotinic acid hydrazide) or only DNA repair (mixture of penicillin G and procaine penicillin G) or both (cyclophosphamide, phenylbutazone, procarbazine, nalidixic acid).

Vincristine shows no effect on the thymidine incorporation in DNA, but by density gradient centrifugation it has been found that it influences the ligase reaction.

Two DNA polymerases had been isolated from spleen cells, one of the low molecular and one of the high molecular weight type. The influences of the described drugs on these enzymes and on a deoxyribonuclease I from beef pancreas have also been tested in *in vitro* systems. In all cases, it has been found that there is no effect or only a very small one, compared with the action of well-known inhibitors as *e.g.* ethidium bromide and p-chloromercuribenzoate, and this cannot be responsible for the suppressions found in DNA repair and semiconservative synthesis.